# Supplementary material for: Deep learning approaches show promise for predicting childhood malnutrition: A comparative study with traditional machine learning methods using survey data
Source: PLoS One. 2026 Jul 22;21(7):e0353969. doi: 10.1371/journal.pone.0353969 (PMC13390942; doi:10.1371/journal.pone.0353969)
Supplement: S1 Appendix — (PDF) [file pone.0353969.s001.pdf]

Appendix

Separability

Following (Figure 8) principal component analysis (PCA), t-distributed stochastic neighbor embedding (t-SNE), and uniform manifold approximation and projection (UMAP) shows that the features used in the model are not fully sufficient to easily distinguish between nourished and malnourished individuals. Therefore, we employed sophisticated machine learning and deep learning algorithms.

Performance Profile

Figure 9 presents a comparative performance profile across model categories using six key metrics. Deep learning models (blue solid line) demonstrate the largest coverage area, achieving the highest scores in recall (0.61), precision (0.61), F1-score (0.61), and balanced accuracy (0.61), while matching other categories in ROC-AUC (0.63). Traditional machine learning methods (green dotted line) and gradient boosting algorithms (orange dashed line) exhibit nearly overlapping profiles, with both reaching ROC-AUC of 0.63-0.64 but showing slightly lower performance in recall and F1-score (approximately 0.60). All three categories converge at similar accuracy levels (0.61), indicating that the performance advantage of deep learning manifests primarily in precision-recall balance and class-specific metrics rather than overall accuracy. The symmetric radar pattern for gradient boosting and traditional machine learning suggests comparable strengths across all evaluation dimensions, while deep learning's expanded polygon reflects more pronounced capability in handling the imbalanced malnutrition classification task.

Hyperparameter and Reproducibility Implementation

To ensure full reproducibility of all reported results, the following measures together with hyperparameter (presented in Table 1) were implemented:

Random Seed Control: A global seed value of 42 was applied across all stochastic components, including Python's built-in `random` module, NumPy, TensorFlow, and PyTorch. This was enforced through `PYTHONHASHSEED=42`, `random.seed(42)`, `np.random.seed(42)`, `tf.random.set_seed(42)`, `tf.keras.utils.set_random_seed(42)`, `torch.manual_seed(42)`, and `torch.cuda.manual_seed_all(42)`. Individual Keras models employed distinct seeds (DNN: 42, ResNet: 43, Wide & Deep: 44) to prevent identical weight initialization across models while maintaining within-model reproducibility.

Deterministic Operations: Environment variables `TF_DETERMINISTIC_OPS=1` and `TF_CUDNN_DETERMINISTIC=1` were set to enforce deterministic GPU operations in TensorFlow. PyTorch determinism was configured through `torch.backends.cudnn.deterministic=True` and `torch.backends.cudnn.benchmark=False`, eliminating non-deterministic algorithmic choices.

Reproducible Data Processing: The train-test split (80:20 ratio) used `random_state=42` with stratified sampling to preserve class proportions across partitions. SMOTE oversampling was applied with `random_state=42` to generate consistent synthetic minority class samples within the training data.

Explicit Weight Initialization: All neural network layers used explicitly seeded initializers – Glorot Uniform for sigmoid and linear output layers, and He Normal for ReLU-based hidden layers in the Wide & Deep architecture. Per-layer seed offsets prevented identical weights across layers within the same model.

Category	Model	Hyperparameters
Traditional ML	Logistic Regression	max_iter=1000, random_state=42, default L2 regularization
	KNN	n_neighbors=5
	LDA	default solver (svd)
	Decision Tree	max_depth=10, random_state=42
	Random Forest	n_estimators=200, max_depth=15, min_samples_split=5, n_jobs=-1, random_state=42
	Extra Trees	n_estimators=300, max_depth=20, min_samples_split=4, min_samples_leaf=2, max_features='sqrt', bootstrap=True, n_jobs=-1, random_state=42
	SVM	kernel='rbf', probability=True, random_state=42
Gradient Boosting	XGBoost	n_estimators=200, max_depth=6, learning_rate=0.05, subsample=0.8, colsample_bytree=0.8, eval_metric='logloss', n_jobs=-1, random_state=42
	LightGBM	n_estimators=300, max_depth=7, learning_rate=0.05, num_leaves=50, subsample=0.8, colsample_bytree=0.8, min_child_samples=20, reg_alpha=0.1, reg_lambda=0.1, n_jobs=-1, random_state=42
	CatBoost	iterations=200, depth=6, learning_rate=0.05, l2_leaf_reg=3, subsample=0.8, random_state=42
	Hist Gradient Boosting	max_iter=200, max_depth=7, learning_rate=0.05, l2_regularization=1.0, random_state=42
	AdaBoost	base_estimator=DecisionTree(max_depth=3), n_estimators=200, learning_rate=0.1, random_state=42
Deep Learning	DNN (Keras)	Architecture: 256$\rightarrow$128$\rightarrow$64$\rightarrow$1; Activation: LeakyReLU(0.1); Optimizer: Adam (lr=0.001); Dropout: 0.3, 0.3, 0.2; L2: 0.001; BatchNorm: yes; Loss: binary_crossentropy; Batch=64; Epochs=100 (patience=20); Initializer: GlorotUniform
	ResNet (Keras)	Architecture: 128$\rightarrow$ResBlock(128)$\rightarrow$ResBlock(64)$\rightarrow$1; Activation: LeakyReLU(0.1); Optimizer: Adam (lr=0.0005); Dropout: 0.3, 0.2; L2: 0.001; BatchNorm: yes; Loss: binary_crossentropy; Batch=64; Epochs=100 (patience=20); Initializer: GlorotUniform
	Wide & Deep (Keras)	Wide: 128$\rightarrow$64 (ReLU); Deep: 384$\rightarrow$192$\rightarrow$96 (LeakyReLU); Combined: 128$\rightarrow$64$\rightarrow$32$\rightarrow$1; Optimizer: Adam (lr=0.001); Dropout: 0.25-0.40; L2: 0.0005-0.001; BatchNorm: yes; Loss: binary_crossentropy; Batch=64; Epochs=100 (patience=20); Initializer: HeNormal
	TabNet (PyTorch)	n_d=64, n_a=64, n_steps=5, gamma=1.5, lambda_sparse=1e-4, momentum=0.02, clip_value=2.0; Optimizer: Adam (lr=0.02, weight_decay=1e-5); Scheduler: StepLR (step_size=50, gamma=0.95); mask_type='sparsemax'; Batch=1024 (virtual=256); Epochs=200 (patience=50)

Table 1. Complete hyperparameter specifications for all 16 machine learning and deep learning models

Feature Selection Ablation Analysis

Table 2 presents the comparative performance of TabNet for strict Boruta-confirmed vs. multi-criteria consensus feature sets. Both feature sets were evaluated using identical preprocessing pipelines, train-test splits (random_state=42), SMOTE oversampling on training data only, and TabNet hyperparameter configurations to ensure fair comparison.

**Metric**	**Boruta-Confirmed**	**Multi-Criteria**	**Difference**
Accuracy	0.60	0.62	+0.02
Precision	0.60	0.63	+0.03
Recall	0.60	0.62	+0.02
F1-Score	0.60	0.62	+0.02
ROC-AUC	0.62	0.64	+0.02
Average Precision	0.53	0.54	+0.01
Balanced Accuracy	0.59	0.62	+0.03
Cohen's Kappa	0.19	0.24	+0.05
MCC	0.19	0.24	+0.05
Brier Score	0.24	0.24	0.00

Table 2. TabNet Performance Comparison

The multi-criteria consensus approach demonstrated consistent improvements over strict Boruta-only selection across all evaluation metrics. The higher performance of the multi-criteria approach validates our methodological choice to retain features that demonstrated strong support across multiple ensemble ranking methods and established domain relevance.
