# Supplementary material for: Deep learning approaches show promise for predicting childhood malnutrition: A comparative study with traditional machine learning methods using survey data
Source: PLoS One. 2026 Jul 22;21(7):e0353969. doi: 10.1371/journal.pone.0353969 (PMC13390942; doi:10.1371/journal.pone.0353969)
Supplement: Attachment — Submitted filename: Response to Reviewers.pdf [file pone.0353969.s003.pdf]

Response to academic editor

We thank the academic editor for the careful evaluation of our manuscript and for highlighting these important methodological points. The first five comments closely align with concerns raised by the reviewers and have been comprehensively addressed in our point-by-point responses below. For clarity, we provide brief cross-references along with a summary of actions taken. The copyright concern regarding Figure 4 is addressed in detail at the end of this section.

1. Clarify the preprocessing and resampling workflow, including whether SMOTE or any balancing procedure was applied.

   Response:

   Addressed in our response to reviewer 2, comment 2.2 (part 1). We have added explicit documentation of the SMOTE oversampling procedure (applied to training data only within cross-validation folds) and the standardized preprocessing pipeline applied across all 16 models in the revised Pre-processing subsection.

2. Provide sufficient implementation details for the deep learning models to ensure reproducibility.

   Response:

   Addressed in our response to reviewer 2, comment 2.2 (part 2). We have added comprehensive hyperparameter tables for all models (including architectures, layer sizes, activation functions, optimizers, learning rates, and regularization) along with a dedicated reproducibility implementation subsection added Appendix [A.3] in our manuscript.

3. Clarify the rationale for the combined malnutrition outcome definition and discuss its implications and limitations.

   Response:

   We have clarified the definition of composite outcome in Section 2.1.2 (Variables and Measurements) and added the rationale by explaining that the composite definition was primarily driven by class imbalance considerations. Individual conditions exhibited substantial imbalance (stunting: 32.37%, underweight: 23.69%, wasting: 11.89%), with wasting being particularly problematic for classification modeling. Combining these into a composite outcome provided a more modeling-friendly distribution (57.2% nourished vs. 42.8% malnourished). Detailed responses are provided in reviewer 1, comment 1.4 (Part 1a) regarding the composite outcome definition and overlap analysis, and reviewer 2, comment 2.3 (Part 2) regarding the acknowledgment of this approach as a limitation in our manuscript.

4. Reconsider or statistically justify claims regarding superiority of specific models where performance differences are marginal.

   Response:

   Addressed in our response to reviewer 2, comment 2.3 (part 1). We have revised the manuscript title from "Deep learning outperforms · · ·" to "Deep learning approaches show promise · · ·", tempered superiority language throughout, and reframed TabNet as being "among a cluster of similarly-performing top models."

5. Address concerns regarding terminology related to dataset balance and interpretation of predictive performance metrics.

   Response:

   Addressed in our responses to reviewer 1 comment 1.5. Regarding dataset balance terminology, we have corrected "balanced" terminology to "moderate class imbalance" throughout the manuscript.
   Regarding interpretation of predictive performance metrics, performance language has been moderated throughout the manuscript and clarified our rationale for prioritizing F1-score and recall as primary metrics in Introduction and Discussion sections: (i) test data retained the original class distribution despite SMOTE-balanced training, requiring imbalance-aware evaluation; (ii) the cost asymmetry of

false negatives (missed malnourished children) substantially exceeds that of false positives; and (iii) F1-score ensures balanced precision-recall performance critical for screening applications.

Journal Requirements:

Copyright concern - Figure 4

Response:

We thank the editor for the careful copyright review of Figure 4. We wish to clarify that Figure 4 was created entirely by the authors using open-source resources fully compatible with the CC BY 4.0 license:

1. Shapefile source: Nepal's administrative boundary shapefile was obtained from geoBoundaries (https://www.geoboundaries.org/), distributed under the CC BY 4.0 license.
2. Data source: Malnutrition prevalence values were derived from Nepal NMICS 2019, the publicly available dataset used throughout our analysis.
3. Software: The figure was generated using Python with open-source GeoPandas and Matplotlib libraries. No proprietary mapping services (Google Maps, Google Earth, Street View) were used.

Since all components are open-source and CC BY 4.0-compatible, no third-party copyright permissions are required. We have updated the Figure 4 caption with citation to include proper attribution as required by the geoBoundaries license as below:

"Figure 4: Spatial distribution of child malnutrition prevalence across all provinces of Nepal. Administrative boundary shapefiles obtained from geoBoundaries [30]; malnutrition prevalence values derived from Nepal NMICS 2019. Figure generated by the authors using Python (GeoPandas and Matplotlib)"

Code sharing concern

Response:

We thank the editor for highlighting PLOS ONE's code sharing guidelines. In compliance with these requirements, we have made all author-generated code publicly available to ensure full reproducibility and facilitate reuse by the research community.

We have modified a Data and Code Availability Statement to the manuscript:

"Python programming language was used for this work and the datasets used in this study are publicly available from the Multiple Indicator Cluster Survey repository (https://mics.unicef.org/surveys). All author-generated code for data preprocessing, feature selection, model training, evaluation, and visualization is publicly available on GitHub (https://github.com/deepakbastola/malnutrition) under the MIT License, ensuring unrestricted access for reproducibility and reuse."

1 Response to reviewer 1

1.1 Firstly, the claim that the study is the "first comprehensive assessment" seems strong given the growing body of literature on child undernutrition. To strengthen this claim, please either document how you established this (e.g., through a brief structured search) or soften the wording to "a comprehensive assessment" or "one of the first applications of ML and DL to identify child malnutrition in Nepal."

Response:

We thank the reviewer for this thoughtful suggestion regarding more appropriate phrasing. We agree that a more measured wording is appropriate to acknowledge the broader literature on child undernutrition. We have revised the manuscript accordingly, replacing the original claim with "one of the first applications of ML and DL to identify child malnutrition in Nepal." This change has been applied consistently throughout the manuscript.

1.2 Secondly, please report the total number of children for each response variable, including stunting, wasting, and underweight.

Response:

We thank the reviewer for this important request. We have added the following sentence in "Variables and measurements" section to clarify the prevalence of each individual response variable in our dataset:
In the data set, 1,520 children (23.69%) were underweight, 2,077 children (32.37%) were stunted, and 763 children (11.89%) were wasted.

1.3 Thirdly, have the authors excluded cases with extreme values (e.g., less than -6 standard deviations or greater than +6) if so, please report this in the paper.

Response:

We thank the reviewer for this important point. We examined all three z-scores for extreme values ($|z-score| > 6$ s.d. per WHO guidelines) and found no cases requiring exclusion. The z-scores lies in the following range:

- HAZ (stunting): range -5.98 to 5.86
- WHZ (wasting): range -5.00 to 5.00
- WAZ (underweight): range -5.62 to 4.47

The absence of extreme values reflects quality control procedures applied during NMICS 2019 data collection and processing by UNICEF and Nepal's Central Bureau of Statistics.

1.4 Fourthly, stunting, wasting, and underweight are related anthropometric constructs. Please clarify how you avoided circularity (e.g., by using components of the outcome definition as predictors) and whether the outcomes were modeled jointly or separately.

The logical definition "any of underweight OR stunted OR wasted" is defensible and commonly used, but the label "balanced" is not statistically accurate.

The individual prevalences (23.37%, 32.37%, and 11.89%) are plausible and align with the fact that stunting is usually more prevalent than wasting.

Given the overlap between these conditions, the combined prevalence of 42% is reasonable. Children who are both wasted and stunted are almost always underweight, which inflates the overlap.

However, calling 58% versus 42% "balanced" is problematic because it still represents a moderately imbalanced binary outcome.

To make the overlap structure transparent, please report a Venn diagram or a small table showing how many children have 0, 1, 2, or 3 conditions.

Response:

We thank the reviewer for raising these important methodological considerations. We address each point below with comprehensive clarifications:

1. We employed two deliberate methodological choices to avoid circularity.
   (a) Anthropometric z-scores excluded as predictors: The composite malnutrition outcome was defined from three anthropometric z-scores (HAZ, WHZ, WAZ). That is, for each child i, the composite binary malnutrition outcome Y_i is defined as,

$$Y_i = \begin{cases} 1, & \text{if } \text{HAZ}_i < -2 \text{ OR } \text{WHZ}_i < -2 \text{ OR } \text{WAZ}_i < -2 \\ 0, & \text{otherwise} \end{cases}$$

   But none of these z-scores, nor their underlying components (height, weight) were included as predictors in any model. This separation ensures that no direct mathematical components of the outcome appear among the predictors, eliminating the most obvious source of circularity. We modeled the composite outcome jointly rather than separately because separate models faced severe imbalance.

   (b) Categorical (not continuous) child age encoding: While child age contributes to HAZ and WAZ calculations, we deliberately operationalized age as a categorical variable (< 1 year, 1 year, 2 years, 3 years, 4 years) with five broad categories rather than continuous age in months.

   This encoding choice substantially mitigates any residual circularity concern for several reasons:

      i. Z-score computation requires precise age in months: HAZ and WAZ use age-specific WHO reference values at monthly intervals (e.g., 14 months vs. 15 months yields different z-score thresholds)

      ii. Broad 12-month categories cannot reconstruct precise z-scores: The substantial within-category variation in actual ages prevents the model from "learning" the z-score formula

      iii. Categories capture developmental stages, not mathematical thresholds: Our age bins reflect biologically meaningful periods (infancy, toddlerhood, preschool years) with distinct nutritional risk profiles independent of z-score calculation

**Age category**	**Developmental phase**	**Independent risk factors**
<1 year	Infancy	Breastfeeding patterns, weaning timing, infection susceptibility
1 year	Toddlerhood	Complementary feeding transition, dietary diversity introduction
2 years	Early childhood	Self-feeding development, food preference establishment
3 years	Preschool	Increased physical activity, varied diet exposure
4 years	Late preschool	School readiness, established eating patterns

Remark: Despite contributing to z-score computation, we retained categorical child age as a predictor because each age category represents a distinct developmental and nutritional phase with characteristic risk profiles. Recent studies have similarly retained age as a predictor in malnutrition prediction models, including Tamanna et al. [5] and Talukder & Ahammed [4] in Bangladesh, Bitew et al. [1] in Ethiopia, and Shen et al. [3] in Papua New Guinea.

Acknowledging the valid reviewers concern, we have added explicit text to the "Variables and Measurements" section which may clarify the readers. "To prevent circularity between outcome definition and predictors, we adopted two methodological safeguards: (1) anthropometric z-scores (HAZ, WHZ, WAZ) and their components (height, weight) were excluded from the predictor set, ensuring no direct mathematical components of the outcome appeared among input features; and (2) child age was operationalized as a categorical variable with five broad categories (< 1, 1, 2, 3, 4 years) rather than continuous age in months. This categorical encoding captures developmentally meaningful stages of

early childhood while deliberately avoiding fine-grained age information that could introduce circularity with HAZ and WAZ calculations, which require precise monthly age for WHO reference comparisons."

2. Class imbalance terminology corrected: We have revised manuscript throughout to: "has moderate class imbalance with 3,671 (57.2%) nourished and 2,745 (42.8%) malnourished children."

3. Following the reviewer's suggestion, we have added the following table making the overlap structure transparent:

**Conditions present**	**n**	**% of total**	**% of malnourished**
No malnutrition	3,671	57.2%	–
Single condition			
Stunted only	982	15.3%	35.8%
Wasted only	243	3.8%	8.9%
Underweight only	123	1.9%	4.5%
Two conditions			
Stunted + Wasted	0	0%	0%
Stunted + Underweight	877	13.7%	31.9%
Wasted + Underweight	302	4.7%	11.0%
Three conditions			
All three (Stunted + Wasted + Underweight)	218	3.4%	7.9%
Total malnourished	2,745	42.8%	100%

Table 1: Overlap structure of malnutrition conditions in the dataset ($n = 6,416$)

1.5 Lastly, at the 3.2 Performance Evaluation mentioned, given the imbalanced dataset, justify why this evaluation is necessary.

Please clarify the methodological objective. Is the main aim to develop a deployable prediction tool, or is it to systematically compare families of algorithms? A clear primary aim will help justify the need for benchmarking 16 models and interpreting the findings. Authors mentioned Domain-informed variable selection, which involved using established literature on child malnutrition determinants and expert consultation. However, they cited paper 30, which revealed that, when combined with the SHAP value method, the optimal prediction model identified living in the Highlands Region, the child's age, family wealth, and birth size as the top five important characteristics for predicting stunting.

It would be more informative if authors explained why they excluded these variables. Alternatively, a reanalysis incorporating these variables would be beneficial, as stunting is a significant form of impaired growth and development in children.

Response:

We thank the reviewer for these insightful comments that help us strengthen the methodological clarity and rigor of our study. We address each point below:

1. Justification for evaluation approach: We acknowledge that our original Section 3.2 did not explicitly justify the evaluation rationale. While the prioritization of F1-score and recall was discussed in the Introduction and Discussion, we recognize that explicit justification within the Performance Evaluation section strengthens methodological clarity. We have added the following text to Section 3.2:

   "Performance evaluation was conducted to enable systematic comparison across families of machine learning and deep learning models. Although SMOTE oversampling was applied to training data (Section 2.1.3), the test set retained the original imbalance class distribution, necessitating imbalance-aware evaluation. We prioritize F1-score and recall due to the cost asymmetry of false negatives and complement these with eight additional metrics (precision, accuracy, ROC-AUC, average precision, balanced accuracy, Cohen's kappa, MCC, Brier score) for comprehensive assessment."

We also recognize that SMOTE application was not adequately documented in our original submission. We have now added explicit description to the Pre-processing section:

"To address the moderate class imbalance, we applied Synthetic Minority Over-sampling Technique (SMOTE) [2] within each cross-validation fold. SMOTE was applied exclusively to training data after the train-test split to prevent data leakage, generating synthetic minority class examples through interpolation. Test data retained the original distribution to ensure evaluation metrics reflected real-world model performance. The preprocessing pipeline was applied uniformly across all 16 machine learning and deep learning models to ensure fair comparison. Feature scaling using StandardScaler (zero mean, unit variance) was additionally applied to models sensitive to feature magnitudes: linear methods (Logistic Regression, LDA), distance-based methods (KNN, SVM), and all neural networks (DNN, ResNet, Wide & Deep). Tree-based models (Decision Tree, Random Forest, Extra Trees), gradient boosting variants (XGBoost, LightGBM, CatBoost, Histogram Gradient Boosting, AdaBoost), and TabNet (which incorporates internal feature processing) used unscaled features, consistent with their algorithmic requirements. StandardScaler was fitted exclusively on training data and applied to test data to prevent data leakage. This standardized pipeline ensures that observed performance differences reflect genuine algorithmic capabilities rather than preprocessing inconsistencies."

2. Methodological objective clarification: The primary aim of this study is systematic methodological comparison of machine learning and deep learning algorithm families for childhood malnutrition identification using survey data, rather than development of a deployment-ready prediction tool. Specifically, we seek to determine: (1) which algorithmic family (deep learning, gradient boosting, or traditional ML) achieves superior performance, (2) whether attention-based architectures (TabNet) offer advantages over established methods, and (3) which determinants emerge as robust predictors across multiple algorithms.

3. Variable selection and reference [30]: We appreciate the opportunity to clarify our variable selection in relation to reference [30]. The key predictors identified in that study were incorporated in our analysis with appropriate contextual adaptation: child age (categorical) and wealth index were directly included as predictors. The "Highlands Region" referenced in [30] is one of the administrative divisions of Papua New Guinea, serving as a geographical indicator specific to that country's context. The analogous administrative divisions in Nepal are the seven provinces (Koshi, Madhesh, Bagmati, Gandaki, Lumbini, Karnali, and Sudurpashchim), which were included in our models to capture geographic disparities relevant to Nepal. Birth size/weight was unavailable in the NMICS 2019 dataset and could not be incorporated.

2 Response to reviewer 2

2.1 Structural & Editorial Requirements

The inclusion of performance results (e.g., F1-score = 0.62) in the Introduction is inappropriate. These results should be moved to the Results section. The dataset is described as "balanced," but the class distribution (approximately 58% vs 42%) indicates a mild class imbalance. This should be corrected.

Response:

We thank the reviewer for these valuable observations. We have addressed both points as follows:

1. We agree and revise the Introduction section to remove all numerical performance metrics, retaining only the general findings necessary to convey the study's contribution.
2. We agree the term "balanced" was statistically inaccurate. We have revised throughout to: "moderate class imbalance".

2.2 Methodological Rigor & Reproducibility

It is not clearly stated whether SMOTE or any resampling method was applied only within the training data. If applied before the train-test split, this would introduce data leakage and inflate performance estimates. This must be clarified. The deep learning models lack essential implementation details such as architecture design, layer sizes, activation functions, optimizers, and learning rates. This limits reproducibility and should be provided. The feature selection process includes variables that were rejected by Boruta but still retained based on ensemble ranking and domain knowledge. This requires clearer justification, preferably supported by sensitivity or ablation analysis. The Brier score is mentioned but not properly defined in the Methods section and should be explicitly included. It is unclear whether consistent preprocessing steps (normalization, scaling, imputation) were applied across both machine learning and deep learning models.

Response:

We thank the reviewer for these constructive comments aimed at improving methodological transparency and reproducibility. We address each point below:

1. SMOTE: We recognize that SMOTE application was not adequately documented in our original submission except flow chart (figure 1). We have now added explicit description to the Pre-processing section: "Class Imbalance Handling: To address the moderate class imbalance, we applied Synthetic Minority Over-sampling Technique (SMOTE) [2] within each cross-validation fold. SMOTE was applied exclusively to training data after the train-test split to prevent data leakage, generating synthetic minority class examples through interpolation. Test data retained the original distribution to ensure evaluation metrics reflected real-world model performance."
2. Reproducibility: Detailed hyperparameter tables for all 16 models (traditional ML, gradient boosting, and deep learning) have been added to Appendix [A.3], including layer architectures, activation functions, optimizers, learning rates, regularization parameters, batch sizes, and training configurations. Reproducibility Implementation subsection added to the Appendix documenting random seed control across Python, NumPy, TensorFlow, and PyTorch (seed=42), deterministic GPU operations, reproducible data preprocessing, and explicit weight initialization with seeded initializers.
3. Ablation analysis: Following the reviewer's suggestion, we conducted a formal ablation analysis using TabNet to empirically validate this feature selection strategy. We compared two feature sets: (1) strict Boruta-confirmed features only, and (2) our multi-criteria consensus selection. Results demonstrate consistent performance improvements with the multi-criteria approach across all evaluation metrics. The complete ablation analysis with detailed performance comparisons has been added to Appendix [A.4], providing empirical justification for our feature selection methodology. We also added the text "The suitability of this multi-criteria feature selection strategy was empirically validated through a formal ablation analysis comparing our approach against strict Boruta-confirmed-only selection, with detailed results presented in Appendix A.4. The ablation analysis confirmed that retaining these

domain-relevant features improved model performance across all evaluation metrics compared to strict Boruta-only selection." in the subsection (2.2.2 Final Feature).

4. Brier Score definition: We confirm that the Brier score has been defined under subsection 2.4 Model Evaluation of section 2 Materials and Methods in the manuscript.

5. Preprocessing steps: We have added the following clarification to explicitly verify identical preprocessing was applied across machine learning and deep learning models:

   "The preprocessing pipeline was applied uniformly across all 16 machine learning and deep learning models to ensure fair comparison. Feature scaling using StandardScaler (zero mean, unit variance) was additionally applied to models sensitive to feature magnitudes: linear methods (Logistic Regression, LDA), distance-based methods (KNN, SVM), and all neural networks (DNN, ResNet, Wide & Deep). Tree-based models (Decision Tree, Random Forest, Extra Trees), gradient boosting variants (XGBoost, LightGBM, CatBoost, Histogram Gradient Boosting, AdaBoost), and TabNet (which incorporates internal feature processing) used unscaled features, consistent with their algorithmic requirements. StandardScaler was fitted exclusively on training data and applied to test data to prevent data leakage. This standardized pipeline ensures that observed performance differences reflect genuine algorithmic capabilities rather than preprocessing inconsistencies."

2.3 Results & Statistical Validity

Claims that TabNet "outperforms" other models are not statistically supported, as performance differences are very small (e.g., F1 = 0.62 vs 0.61). Statistical testing (e.g., McNemar's test or bootstrap confidence intervals) is required to support such claims. Cohen's Kappa values (0.23) indicate only fair agreement. Therefore, describing the models as strong or highly effective is an overstatement. Some variables, particularly "Meal Frequency," appear to contain data collection inconsistencies or proxy responses. Retaining these without adjustment may affect model reliability. Combining underweight, stunting, and wasting into a single binary outcome hides important clinical differences and should be explicitly acknowledged as a limitation.

Response:

We thank the reviewer for these constructive comments that strengthen the methodological rigor and interpretation of our findings. We address each point below:

1. We agree that the small numerical differences between top-performing models do not warrant strong claims of superiority without formal statistical testing. We have substantially tempered our language throughout the manuscript to accurately reflect the modest magnitude of these differences.

   (a) Revised manuscript title: We have revised the title to better reflect our findings:

      Original: "Deep learning outperforms traditional machine learning methods in predicting childhood malnutrition: evidence from survey data"

      Revised: "Deep learning approaches show promise for predicting childhood malnutrition: a comparative study with traditional machine learning methods using survey data"

   (b) Revised language throughout manuscript,

Original	Revised
TabNet outperforms	TabNet achieved the highest scores
superior performance	strong performance
outperforms traditional ML	marginal performance advantages over traditional ML
best-performing model	top-ranked model

2. Composite Outcome Limitation: We agree to reviewer suggestion and added the following paragraph in the Limitation section.

"Combining stunting, wasting, and underweight into a single binary outcome obscures clinically important distinctions between underweight, stunting, and wasting, which require different intervention strategies. Our composite approach suits population-level screening but limits clinical actionability for intervention type selection. Future research should develop condition-specific prediction models to enable more targeted clinical decisions."

2.4 Discussion & Recommendations

The claim that deep learning significantly outperforms traditional machine learning is not strongly supported by the results. Overall performance appears comparable across model classes. Policy recommendations are too strong given the modest predictive performance (Kappa ≈ 0.23). These should be toned down and presented more cautiously. Comparisons with other studies (e.g., Ethiopia, Philippines, Papua New Guinea) should explicitly account for differences in sample size, feature sets, outcome definitions, and evaluation metrics to avoid misleading interpretation.

Response:

We thank the reviewer for these valuable observations that help us calibrate our claims more appropriately to the evidence. We address each point below:

1. We agree that our original framing overstated the performance advantages of deep learning approaches. We have systematically revised the manuscript including the title to present findings more accurately, comprehensively mentioned in part III above.

2. Policy recommendations are carefully revised and softened in Recommendation subsection (under section 4 Discussion).

3. Sample size: Sample size variations across studies are explicitly documented in Table 4 (Comparative summary across existing literature).

   Feature sets: We have added clarification regarding our feature selection approach in relation to prior studies: "Our initial candidate features were informed by established work in similar contexts, including studies from Bangladesh, India, Ethiopia, Philippines, and Papua New Guinea. After applying our multi-criteria feature selection pipeline (detailed in section 2.1.2 Variables and Measurements), the final feature set included commonly identified determinants across countries was child age, wealth index, maternal education, and geographic region. However, novel features specific to our dataset such as away privileges and left alone, which captured child supervision patterns not consistently examined in prior work. This shared set of features indicates the presence of common underlying etiological pathways for childhood malnutrition, whereas the context-specific features primarily capture variation arising from differences in information availability across datasets."

   Outcome definitions: We have explicitly documented the variation in outcome definitions across studies in Table 4 and added the following text (in section 4 Discussion). "Outcome definitions vary substantially across studies. Some studies focus on single anthropometric indicators (stunting only in [10,11]), others examine all three indicators separately (stunting, wasting, and underweight as distinct outcomes), and [35] use composite indicators similar to our approach as docu- mented in Table 4."

   Evaluation metrics: We have substantially tempered our language throughout the manuscript to accurately reflect evaluation performance and corrected Cohen's Kappa interpretation as "fair agreement" (0.24) rather than strong performance. Acknowledged absence of formal statistical testing as a limitation, noting that observed performance differences (typically 1-3 percentage points) cannot be definitively characterized as statistically significant without formal tests.

References

[1] Fikrewold H Bitew, Corey S Sparks, and Samuel H Nyarko. Machine learning algorithms for predicting undernutrition among under-five children in Ethiopia. *Public Health Nutrition*, 25(2):269–280, 2022.

[2] Nitesh V Chawla, Kevin W Bowyer, Lawrence O Hall, and W Philip Kegelmeyer. Smote: synthetic minority over-sampling technique. *Journal of Artificial Intelligence Research*, 16:321–357, 2002.

[3] Hao Shen, Hang Zhao, and Yi Jiang. Machine learning algorithms for predicting stunting among under-five children in Papua New Guinea. *Children*, 10(10), 2023.

[4] Ashis Talukder and Benojir Ahammed. Machine learning algorithms for predicting malnutrition among under-five children in Bangladesh. *Nutrition*, 78:110861, 2020.

[5] Tanzila Tamanna, Shohel Mahmud, Nahid Salma, Md. Musharraf Hossain, and Md. Rezaul Karim. Identifying determinants of malnutrition in under-five children in Bangladesh: insights from the BDHS-2022 cross-sectional study. *Scientific Reports*, 15(1):14336, 2025.
