# Supplementary material for: Deep learning approaches show promise for predicting childhood malnutrition: A comparative study with traditional machine learning methods using survey data
Source: PLoS One. 2026 Jul 22;21(7):e0353969. doi: 10.1371/journal.pone.0353969 (PMC13390942; doi:10.1371/journal.pone.0353969)
Supplement: Attachment — Submitted filename: Response to Reviewers.docx [file pone.0353969.s004.docx]

Thank you for considering the second revision of our manuscript and for providing us with the opportunity to address the remaining comments. We have revised the manuscript to address all the minor comments, as detailed below. We sincerely appreciate the constructive feedback provided, which has helped us improve the quality and accuracy of our manuscript.

**Response to Academic Editor**

**Comment:** In both the main text and the table captions, *Table .5.* and *Table .6.* are incorrectly formatted. Please remove the unnecessary periods before the table numbers so that they appear as *Table 5* and *Table 6*, respectively.

**Response:** Thank you for pointing out this formatting issue. We have corrected the table references in both the main text and the table captions. They now appear correctly as *Table 5* and *Table 6*.

**Response to Reviewer # 2**

**Comment 1:** In lines 405-406, the text states that SVM matches TabNet's recall. However, Table 3 shows a marginal difference (0.61 vs. 0.62). Please change “matching” to “nearly matching” or “comparable” for absolute precision.

**Response:** Thank you for identifying this wording issue. We have revised the text by replacing **“matching”** with **“nearly matching”** to accurately reflect the results presented in Table 3.

**Comment 2:** Fix the typo in lines 417–418: “K-nearest eighbors” should be “K-nearest neighbors”.

**Response:** Thank you for identifying the typographical error. It has been corrected in the revised manuscript.
