# Supplementary material for: Deep learning approaches show promise for predicting childhood malnutrition: A comparative study with traditional machine learning methods using survey data
Source: PLoS One. 2026 Jul 22;21(7):e0353969. doi: 10.1371/journal.pone.0353969 (PMC13390942; doi:10.1371/journal.pone.0353969)
Supplement: Peer review history

# Peer review: Decision Letter 0

**DOI:** 10.1371/journal.pone.0353969.r001

**Authors:** Merve Kaşıkcı

8 May 2026

PONE-D-26-12244

Deep learning outperforms traditional machine learning methods in predicting childhood malnutrition: evidence from survey data

PLOS One

Dear Dr. Bastola,

Thank you for submitting your manuscript to PLOS ONE. After careful consideration, we feel that it has merit but does not fully meet PLOS ONE’s publication criteria as it currently stands. Therefore, we invite you to submit a revised version of the manuscript that addresses the points raised during the review process.

The manuscript has now been evaluated by two independent reviewers. Both reviewers acknowledge the potential relevance of the study; however, they also raised several important methodological, statistical, and reporting concerns that should be addressed before the manuscript can be considered further.

In particular, the revised manuscript should:

• Clarify the preprocessing and resampling workflow, including whether SMOTE or any balancing procedure was applied.

• Provide sufficient implementation details for the deep learning models to ensure reproducibility.

• Clarify the rationale for the combined malnutrition outcome definition and discuss its implications and limitations.

• Reconsider or statistically justify claims regarding superiority of specific models where performance differences are marginal.

• Address concerns regarding terminology related to dataset balance and interpretation of predictive performance metrics.

Based on the reviewers’ evaluations and my assessment, I am inviting you to submit a major revision of the manuscript for further consideration.

Please submit your revised manuscript by Jun 22 2026 11:59PM. If you will need more time than this to complete your revisions, please reply to this message or contact the journal office at plosone@plos.org. When you're ready to submit your revision, log on to https://www.editorialmanager.com/pone/ and select the 'Submissions Needing Revision' folder to locate your manuscript file.

Please include the following items when submitting your revised manuscript:

- A letter that responds to each point raised by the academic editor and reviewer(s). You should upload this letter as a separate file labeled 'Response to Reviewers'.
- A marked-up copy of your manuscript that highlights changes made to the original version. You should upload this as a separate file labeled 'Revised Manuscript with Track Changes'.
- An unmarked version of your revised paper without tracked changes. You should upload this as a separate file labeled 'Manuscript'.

If you would like to make changes to your financial disclosure, please include your updated statement in your cover letter. Guidelines for resubmitting your figure files are available below the reviewer comments at the end of this letter.

If applicable, we recommend that you deposit your laboratory protocols in protocols.io to enhance the reproducibility of your results. Protocols.io assigns your protocol its own identifier (DOI) so that it can be cited independently in the future. For instructions see: https://journals.plos.org/plosone/s/submission-guidelines#loc-laboratory-protocols. Additionally, PLOS ONE offers an option for publishing peer-reviewed Lab Protocol articles, which describe protocols hosted on protocols.io. Read more information on sharing protocols at https://plos.org/protocols?utm_medium=editorial-email&utm_source=authorletters&utm_campaign=protocols.

As the corresponding author, your ORCID iD is verified in the submission system and will appear in the published article. PLOS supports the use of ORCID, and we encourage all coauthors to register for an ORCID iD and use it as well. Please encourage your coauthors to verify their ORCID iD within the submission system before final acceptance, as unverified ORCID iDs will not appear in the published article. *Only*  the individual author can complete the verification step; PLOS staff *cannot*  verify ORCID iDs on behalf of authors.

We look forward to receiving your revised manuscript.

Kind regards,

Merve Kaşıkcı

Academic Editor

PLOS One

Journal Requirements:

When submitting your revision, we need you to address these additional requirements.

1. Please ensure that your manuscript meets PLOS ONE's style requirements, including those for file naming. The PLOS ONE style templates can be found at

https://journals.plos.org/plosone/s/file?id=wjVg/PLOSOne_formatting_sample_main_body.pdf and

https://journals.plos.org/plosone/s/file?id=ba62/PLOSOne_formatting_sample_title_authors_affiliations.pdf.

2. Please note that PLOS One has specific guidelines on code sharing for submissions in which author-generated code underpins the findings in the manuscript. In these cases, we expect all author-generated code to be made available without restrictions upon publication of the work. Please review our guidelines at https://journals.plos.org/plosone/s/materials-and-software-sharing#loc-sharing-code and ensure that your code is shared in a way that follows best practice and facilitates reproducibility and reuse.

3. Please update your submission to use the PLOS LaTeX template. The template and more information on our requirements for LaTeX submissions can be found at http://journals.plos.org/plosone/s/latex.

4.We note that Figure 4 in your submission contain [map/satellite] images which may be copyrighted. All PLOS content is published under the Creative Commons Attribution License (CC BY 4.0), which means that the manuscript, images, and Supporting Information files will be freely available online, and any third party is permitted to access, download, copy, distribute, and use these materials in any way, even commercially, with proper attribution. For these reasons, we cannot publish previously copyrighted maps or satellite images created using proprietary data, such as Google software (Google Maps, Street View, and Earth). For more information, see our copyright guidelines: http://journals.plos.org/plosone/s/licenses-and-copyright.

We require you to either (1) present written permission from the copyright holder to publish these figures specifically under the CC BY 4.0 license, or (2) remove the figures from your submission:

a. You may seek permission from the original copyright holder of Figure 4 to publish the content specifically under the CC BY 4.0 license.

We recommend that you contact the original copyright holder with the Content Permission Form (http://journals.plos.org/plosone/s/file?id=7c09/content-permission-form.pdf) and the following text:

“I request permission for the open-access journal PLOS ONE to publish XXX under the Creative Commons Attribution License (CCAL) CC BY 4.0 (http://creativecommons.org/licenses/by/4.0/). Please be aware that this license allows unrestricted use and distribution, even commercially, by third parties. Please reply and provide explicit written permission to publish XXX under a CC BY license and complete the attached form.”

Please upload the completed Content Permission Form or other proof of granted permissions as an "Other" file with your submission.

In the figure caption of the copyrighted figure, please include the following text: “Reprinted from [ref] under a CC BY license, with permission from [name of publisher], original copyright [original copyright year].”

b. If you are unable to obtain permission from the original copyright holder to publish these figures under the CC BY 4.0 license or if the copyright holder’s requirements are incompatible with the CC BY 4.0 license, please either i) remove the figure or ii) supply a replacement figure that complies with the CC BY 4.0 license. Please check copyright information on all replacement figures and update the figure caption with source information. If applicable, please specify in the figure caption text when a figure is similar but not identical to the original image and is therefore for illustrative purposes only.

The following resources for replacing copyrighted map figures may be helpful:

USGS National Map Viewer (public domain): http://viewer.nationalmap.gov/viewer/

The Gateway to Astronaut Photography of Earth (public domain): http://eol.jsc.nasa.gov/sseop/clickmap/

Maps at the CIA (public domain): https://www.cia.gov/library/publications/the-world-factbook/index.html and https://www.cia.gov/library/publications/cia-maps-publications/index.html

NASA Earth Observatory (public domain): http://earthobservatory.nasa.gov/

Landsat: http://landsat.visibleearth.nasa.gov/

USGS EROS (Earth Resources Observatory and Science (EROS) Center) (public domain): http://eros.usgs.gov/#

Natural Earth (public domain): http://www.naturalearthdata.com

5. If the reviewer comments include a recommendation to cite specific previously published works, please review and evaluate these publications to determine whether they are relevant and should be cited. There is no requirement to cite these works unless the editor has indicated otherwise.

Reviewers' comments:

Reviewer's Responses to Questions

**Comments to the Author**

1. Is the manuscript technically sound, and do the data support the conclusions?

The manuscript must describe a technically sound piece of scientific research with data that supports the conclusions. Experiments must have been conducted rigorously, with appropriate controls, replication, and sample sizes. The conclusions must be drawn appropriately based on the data presented.

Reviewer #1: Yes

Reviewer #2: No

**********

2. Has the statistical analysis been performed appropriately and rigorously?

Reviewer #1: Yes

Reviewer #2: No

**********

3. Have the authors made all data underlying the findings in their manuscript fully available?

The PLOS Data policy requires authors to make all data underlying the findings described in their manuscript fully available without restriction, with rare exception (please refer to the Data Availability Statement in the manuscript PDF file). The data should be provided as part of the manuscript or its supporting information, or deposited to a public repository. For example, in addition to summary statistics, the data points behind means, medians and variance measures should be available. If there are restrictions on publicly sharing data—e.g. participant privacy or use of data from a third party—those must be specified.

Reviewer #1: Yes

Reviewer #2: Yes

**********

4. Is the manuscript presented in an intelligible fashion and written in standard English?

PLOS ONE does not copyedit accepted manuscripts, so the language in submitted articles must be clear, correct, and unambiguous. Any typographical or grammatical errors should be corrected at revision, so please note any specific errors here.

Reviewer #1: Yes

Reviewer #2: No

**********

5. Review Comments to the Author

Please use the space provided to explain your answers to the questions above. You may also include additional comments for the author, including concerns about dual publication, research ethics, or publication ethics. (Please upload your review as an attachment if it exceeds 20,000 characters)

Reviewer #1: Thank you for your work and the opportunity to review the paper. I have a few points regarding the methodology aspects.

Firstly, the claim that the study is the “first comprehensive assessment” seems strong given the growing body of literature on child undernutrition. To strengthen this claim, please either document how you established this (e.g., through a brief structured search) or soften the wording to “a comprehensive assessment” or “one of the first applications of ML and DL to identify child malnutrition in Nepal.”

Secondly, please report the total number of children for each response variable, including stunting, wasting, and underweight.

Thirdly, have the authors excluded cases with extreme values (e.g., less than -6 standard deviations or greater than +6) if so, please report this in the paper.

Fourthly, stunting, wasting, and underweight are related anthropometric constructs. Please clarify how you avoided circularity (e.g., by using components of the outcome definition as predictors) and whether the outcomes were modeled jointly or separately.

The logical definition “any of underweight OR stunted OR wasted” is defensible and commonly used, but the label “balanced” is not statistically accurate.

The individual prevalences (23.37%, 32.37%, and 11.89%) are plausible and align with the fact that stunting is usually more prevalent than wasting.

Given the overlap between these conditions, the combined prevalence of 42% is reasonable. Children who are both wasted and stunted are almost always underweight, which inflates the overlap.

However, calling 58% versus 42% “balanced” is problematic because it still represents a moderately imbalanced binary outcome.

To make the overlap structure transparent, please report a Venn diagram or a small table showing how many children have 0, 1, 2, or 3 conditions.

Lastly, at the 3.2 Performance Evaluation mentioned, given the imbalanced dataset, justify why this evaluation is necessary.

Please clarify the methodological objective. Is the main aim to develop a deployable prediction tool, or is it to systematically compare families of algorithms? A clear primary aim will help justify the need for benchmarking 16 models and interpreting the findings.

Authors mentioned Domain-informed variable selection, which involved using established literature on child malnutrition determinants and expert consultation. However, they cited paper 30, which revealed that, when combined with the SHAP value method, the optimal prediction model identified living in the Highlands Region, the child’s age, family wealth, and birth size as the top five important characteristics for predicting stunting.

It would be more informative if authors explained why they excluded these variables. Alternatively, a reanalysis incorporating these variables would be beneficial, as stunting is a significant form of impaired growth and development in children.

Reviewer #2: Dear Authors,

Following a detailed review of your manuscript, several important methodological, statistical, and interpretative issues must be addressed before the paper can be considered further.

I. Structural & Editorial Requirements

The inclusion of performance results (e.g., F1-score = 0.62) in the Introduction is inappropriate. These results should be moved to the Results section.

The dataset is described as “balanced,” but the class distribution (approximately 58% vs 42%) indicates a mild class imbalance. This should be corrected.

II. Methodological Rigor & Reproducibility

It is not clearly stated whether SMOTE or any resampling method was applied only within the training data. If applied before the train-test split, this would introduce data leakage and inflate performance estimates. This must be clarified.

The deep learning models lack essential implementation details such as architecture design, layer sizes, activation functions, optimizers, and learning rates. This limits reproducibility and should be provided.

The feature selection process includes variables that were rejected by Boruta but still retained based on ensemble ranking and domain knowledge. This requires clearer justification, preferably supported by sensitivity or ablation analysis.

The Brier score is mentioned but not properly defined in the Methods section and should be explicitly included.

It is unclear whether consistent preprocessing steps (normalization, scaling, imputation) were applied across both machine learning and deep learning models.

III. Results & Statistical Validity

Claims that TabNet “outperforms” other models are not statistically supported, as performance differences are very small (e.g., F1 = 0.62 vs 0.61). Statistical testing (e.g., McNemar’s test or bootstrap confidence intervals) is required to support such claims.

Cohen’s Kappa values (~0.23) indicate only fair agreement. Therefore, describing the models as strong or highly effective is an overstatement.

Some variables, particularly “Meal Frequency,” appear to contain data collection inconsistencies or proxy responses. Retaining these without adjustment may affect model reliability.

Combining underweight, stunting, and wasting into a single binary outcome hides important clinical differences and should be explicitly acknowledged as a limitation.

IV. Discussion & Recommendations

The claim that deep learning significantly outperforms traditional machine learning is not strongly supported by the results. Overall performance appears comparable across model classes.

Policy recommendations are too strong given the modest predictive performance (Kappa ≈ 0.23). These should be toned down and presented more cautiously.

Comparisons with other studies (e.g., Ethiopia, Philippines, Papua New Guinea) should explicitly account for differences in sample size, feature sets, outcome definitions, and evaluation metrics to avoid misleading interpretation.

To ensure your figures meet our technical requirements, please review our figure guidelines: https://journals.plos.org/plosone/s/figures

You may also use PLOS’s free figure tool, NAAS, to help you prepare publication quality figures: https://journals.plos.org/plosone/s/figures#loc-tools-for-figure-preparation.

NAAS will assess whether your figures meet our technical requirements by comparing each figure against our figure specifications.

# Author comment: Author response to Decision Letter 1

**DOI:** 10.1371/journal.pone.0353969.r002

19 May 2026

Corrections are uploaded with separate file with label 'Response to Reviewer'.

Attachment Submitted filename: Response to Reviewers.pdf

# Peer review: Decision Letter 1

**DOI:** 10.1371/journal.pone.0353969.r003

**Authors:** Merve Kaşıkcı, Merve Kaşıkcı

30 Jun 2026

PONE-D-26-12244R1

Deep learning approaches show promise for predicting childhood malnutrition: a comparative study with traditional machine learning methods using survey data

PLOS One

Dear Dr. Bastola,

Thank you for submitting your manuscript to PLOS ONE. After careful consideration, we feel that it has merit but does not fully meet PLOS ONE’s publication criteria as it currently stands. Therefore, we invite you to submit a revised version of the manuscript that addresses the points raised during the review process.

Please submit your revised manuscript by Aug 14 2026 11:59PM. If you will need more time than this to complete your revisions, please reply to this message or contact the journal office at plosone@plos.org. When you're ready to submit your revision, log on to https://www.editorialmanager.com/pone/ and select the 'Submissions Needing Revision' folder to locate your manuscript file.

Please include the following items when submitting your revised manuscript:

- A letter that responds to each point raised by the academic editor and reviewer(s). You should upload this letter as a separate file labeled 'Response to Reviewers'.
- A marked-up copy of your manuscript that highlights changes made to the original version. You should upload this as a separate file labeled 'Revised Manuscript with Track Changes'.
- An unmarked version of your revised paper without tracked changes. You should upload this as a separate file labeled 'Manuscript'.

If you would like to make changes to your financial disclosure, please include your updated statement in your cover letter. Guidelines for resubmitting your figure files are available below the reviewer comments at the end of this letter.

If applicable, we recommend that you deposit your laboratory protocols in protocols.io to enhance the reproducibility of your results. Protocols.io assigns your protocol its own identifier (DOI) so that it can be cited independently in the future. For instructions see: https://journals.plos.org/plosone/s/submission-guidelines#loc-laboratory-protocols. Additionally, PLOS ONE offers an option for publishing peer-reviewed Lab Protocol articles, which describe protocols hosted on protocols.io. Read more information on sharing protocols at https://plos.org/protocols?utm_medium=editorial-email&utm_source=authorletters&utm_campaign=protocols.

As the corresponding author, your ORCID iD is verified in the submission system and will appear in the published article. PLOS supports the use of ORCID, and we encourage all coauthors to register for an ORCID iD and use it as well. Please encourage your coauthors to verify their ORCID iD within the submission system before final acceptance, as unverified ORCID iDs will not appear in the published article. *Only*  the individual author can complete the verification step; PLOS staff *cannot*  verify ORCID iDs on behalf of authors.

We look forward to receiving your revised manuscript.

Kind regards,

Merve Kaşıkcı

Academic Editor

PLOS One

Journal Requirements:

If the reviewer comments include a recommendation to cite specific previously published works, please review and evaluate these publications to determine whether they are relevant and should be cited. There is no requirement to cite these works unless the editor has indicated otherwise.

Please review your reference list to ensure that it is complete and correct. If you have cited papers that have been retracted, please include the rationale for doing so in the manuscript text, or remove these references and replace them with relevant current references. Any changes to the reference list should be mentioned in the rebuttal letter that accompanies your revised manuscript. If you need to cite a retracted article, indicate the article’s retracted status in the References list and also include a citation and full reference for the retraction notice.

**Additional Editor Comments:**

Dear Authors,

Thank you for submitting your revised manuscript.

After editorial evaluation, a few minor issues remain that should be addressed before the manuscript can be accepted for publication:

In both the main text and the table captions, Table .5. and Table .6. are incorrectly formatted. Please remove the unnecessary periods before the table numbers so that they appear as Table 5 and Table 6, respectively. Please ensure that all comments and suggestions provided by Reviewer 2 have been fully addressed in the revised manuscript.

Once these minor revisions have been completed satisfactorily, the manuscript can be accepted for publication.

We look forward to receiving your revised manuscript.

[Note: HTML markup is below. Please do not edit.]

Reviewers' comments:

Reviewer's Responses to Questions

**Comments to the Author**

1. If the authors have adequately addressed your comments raised in a previous round of review and you feel that this manuscript is now acceptable for publication, you may indicate that here to bypass the “Comments to the Author” section, enter your conflict of interest statement in the “Confidential to Editor” section, and submit your "Accept" recommendation.

Reviewer #1: All comments have been addressed

Reviewer #2: All comments have been addressed

**********

2. Is the manuscript technically sound, and do the data support the conclusions?

The manuscript must describe a technically sound piece of scientific research with data that supports the conclusions. Experiments must have been conducted rigorously, with appropriate controls, replication, and sample sizes. The conclusions must be drawn appropriately based on the data presented.

Reviewer #1: Yes

Reviewer #2: Yes

**********

3. Has the statistical analysis been performed appropriately and rigorously?

Reviewer #1: Yes

Reviewer #2: Yes

**********

4. Have the authors made all data underlying the findings in their manuscript fully available?

The PLOS Data policy requires authors to make all data underlying the findings described in their manuscript fully available without restriction, with rare exception (please refer to the Data Availability Statement in the manuscript PDF file). The data should be provided as part of the manuscript or its supporting information, or deposited to a public repository. For example, in addition to summary statistics, the data points behind means, medians and variance measures should be available. If there are restrictions on publicly sharing data—e.g. participant privacy or use of data from a third party—those must be specified.

Reviewer #1: Yes

Reviewer #2: Yes

**********

5. Is the manuscript presented in an intelligible fashion and written in standard English?

PLOS ONE does not copyedit accepted manuscripts, so the language in submitted articles must be clear, correct, and unambiguous. Any typographical or grammatical errors should be corrected at revision, so please note any specific errors here.

Reviewer #1: Yes

Reviewer #2: Yes

**********

6. Review Comments to the Author

Please use the space provided to explain your answers to the questions above. You may also include additional comments for the author, including concerns about dual publication, research ethics, or publication ethics. (Please upload your review as an attachment if it exceeds 20,000 characters)

Reviewer #1: (No Response)

Reviewer #2: Comments to the Authors:

Thank you for comprehensively addressing all the previous comments. The addition of the detailed hyperparameter configurations (Table 5) and the ablation analysis (Table 6) has significantly improved the quality and reproducibility of the manuscript.

Please address these final minor editorial points before publication:

1. In lines 405-406, the text states that SVM matches TabNet's recall. However, Table 3 shows a marginal difference (0.61 vs 0.62). Please change "matching" to "nearly matching" or "comparable" for absolute precision.

2. Fix the typo in lines 417-418: "K-nearest eighbors" should be "K-nearest neighbors".

The manuscript can be considered for publication after fixing these minor issues.

**********

7. PLOS authors have the option to publish the peer review history of their article (what does this mean?). If published, this will include your full peer review and any attached files.

If you choose “no”, your identity will remain anonymous but your review may still be made public.

**Do you want your identity to be public for this peer review?**  For information about this choice, including consent withdrawal, please see our Privacy Policy.

Reviewer #1: No

Reviewer #2: **Yes:** Md. Abdur Rahman Bijoy

**********

[NOTE: If reviewer comments were submitted as an attachment file, they will be attached to this email and accessible via the submission site. Please log into your account, locate the manuscript record, and check for the action link "View Attachments". If this link does not appear, there are no attachment files.]

To ensure your figures meet our technical requirements, please review our figure guidelines: https://journals.plos.org/plosone/s/figures

You may also use PLOS’s free figure tool, NAAS, to help you prepare publication quality figures: https://journals.plos.org/plosone/s/figures#loc-tools-for-figure-preparation.

NAAS will assess whether your figures meet our technical requirements by comparing each figure against our figure specifications.

# Author comment: Author response to Decision Letter 2

**DOI:** 10.1371/journal.pone.0353969.r004

30 Jun 2026

Response to Academic Editor

Comment: In both the main text and the table captions, Table .5. and Table .6. are incorrectly formatted. Please remove the unnecessary periods before the table numbers so that they appear as Table 5 and Table 6, respectively.

Response: Thank you for pointing out this formatting issue. We have corrected the table references in both the main text and the table captions. They now appear correctly as Table 5 and Table 6.

Response to Reviewer # 2

Comment 1: In lines 405-406, the text states that SVM matches TabNet's recall. However, Table 3 shows a marginal difference (0.61 vs. 0.62). Please change “matching” to “nearly matching” or “comparable” for absolute precision.

Response: Thank you for identifying this wording issue. We have revised the text by replacing “matching” with “nearly matching” to accurately reflect the results presented in Table 3.

Comment 2: Fix the typo in lines 417–418: “K-nearest eighbors” should be “K-nearest neighbors”.

Response: Thank you for identifying the typographical error. It has been corrected in the revised manuscript.

Note: Corrections are uploaded with separate file with label 'Response to Reviewers' as well.

Attachment Submitted filename: Response to Reviewers.docx

# Editor report: Decision Letter 2

**DOI:** 10.1371/journal.pone.0353969.r005

**Authors:** Merve Kaşıkcı, Merve Kaşıkcı, Merve Kaşıkcı

1 Jul 2026

Deep learning approaches show promise for predicting childhood malnutrition: a comparative study with traditional machine learning methods using survey data

PONE-D-26-12244R2

Dear Dr. Bastola,

We’re pleased to inform you that your manuscript has been judged scientifically suitable for publication and will be formally accepted for publication once it meets all outstanding technical requirements.

Within one week, you’ll receive an e-mail detailing the required amendments. When these have been addressed, you’ll receive a formal acceptance letter and your manuscript will be scheduled for publication.

An invoice will be generated when your article is formally accepted. Please note, if your institution has a publishing partnership with PLOS and your article meets the relevant criteria, all or part of your publication costs will be covered. Please make sure your user information is up-to-date by logging into Editorial Manager at Editorial Manager® and clicking the ‘Update My Information' link at the top of the page. For questions related to billing, please contact billing support.

If your institution or institutions have a press office, please notify them about your upcoming paper to help maximize its impact. If they’ll be preparing press materials, please inform our press team as soon as possible -- no later than 48 hours after receiving the formal acceptance. Your manuscript will remain under strict press embargo until 2 pm Eastern Time on the date of publication. For more information, please contact onepress@plos.org.

Kind regards,

Merve Kaşıkcı

Academic Editor

PLOS One

# Editor report: Acceptance letter

**DOI:** 10.1371/journal.pone.0353969.r006

**Authors:** Merve Kaşıkcı, Merve Kaşıkcı, Merve Kaşıkcı

PONE-D-26-12244R2

PLOS One

Dear Dr. Bastola,

I'm pleased to inform you that your manuscript has been deemed suitable for publication in PLOS One. Congratulations! Your manuscript is now being handed over to our production team.

At this stage, our production department will prepare your paper for publication. This includes ensuring the following:

* All references, tables, and figures are properly cited

* All relevant supporting information is included in the manuscript submission,

* There are no issues that prevent the paper from being properly typeset

You will receive further instructions from the production team, including instructions on how to review your proof when it is ready. Please keep in mind that we are working through a large volume of accepted articles, so please give us a few days to review your paper and let you know the next and final steps.

Lastly, if your institution or institutions have a press office, please let them know about your upcoming paper now to help maximize its impact. If they'll be preparing press materials, please inform our press team within the next 48 hours. Your manuscript will remain under strict press embargo until 2 pm Eastern Time on the date of publication. For more information, please contact onepress@plos.org.

You will receive an invoice from PLOS for your publication fee after your manuscript has reached the completed accept phase. If you receive an email requesting payment before acceptance or for any other service, this may be a phishing scheme. Learn how to identify phishing emails and protect your accounts at https://explore.plos.org/phishing.

If we can help with anything else, please email us at customercare@plos.org.

Thank you for submitting your work to PLOS ONE and supporting open access.

Kind regards,

PLOS ONE Editorial Office Staff

on behalf of

Dr. Merve Kaşıkcı

Academic Editor

PLOS One
